# Social Media Use and Consumption of Prescription-Free Medications for Anxiety, Sleep, and Pain among Norwegian University Students

**DOI:** 10.3390/ejihpe14080147

**Published:** 2024-08-01

**Authors:** Wajiha Saqib, Parisa Gazerani

**Affiliations:** 1 Department of Life Sciences and Health, Faculty of Health Sciences, Oslo Metropolitan University, 0130 Oslo, Norway; 2 Department of Health Science and Technology, Faculty of Medicine, Aalborg University, 9260 Gistrup, Denmark

**Keywords:** social media, university students, over-the-counter, OTC, medication, prescription-free, pain, anxiety, depression, musculoskeletal pain, headache

## Abstract

A relationship has been recognized between social media use and health issues. However, no studies have explored the potential link between social media use and consumption of over-the-counter (OTC) medications. We examined social media use, self-reported anxiety, depression, sleep problems, pain, and OTC medications use among Norwegian university students. The goal was to gain insights that would guide preventive health strategies for this target group. A quantitative, cross-sectional study was conducted with an online questionnaire distributed to university student Facebook groups in Norway. A total of 132 completed surveys were analyzed. Among the respondents, 28% experienced anxiety, 35% depression, 64% sleep problems, 71% headaches, and 78% musculoskeletal pain. Moreover, 56% reported using OTC analgesics or sleep aids, mostly purchased from community pharmacies. No statistically significant correlation was found between social media use and headache, musculoskeletal pain, sleep disturbances, or consumption of OTC medications among university students in Norway. The findings, however, demonstrated a positive trend, highlighting the need for further research with larger, more diverse samples, and potentially employing a qualitative or longitudinal design. We propose increased awareness of the potential negative effects of social media among university students, the inclusion of social media and health topics in study curricula, and the more proactive engagement of community pharmacists with young clients concerning the consumption of OTC medications.

## 1. Introduction

### 1.1. Social Media and Its Impact

Social media, encompassing websites and apps for content sharing and interaction, have surged globally with technological advancements. In 2023, 4.9 billion people used social media [1], including 88% of Norwegians [2]. Platforms like Facebook, WhatsApp, YouTube, and Instagram have grown rapidly, with the motivations for use varying by factors like gender and life stage [3]. Common motives include entertainment, social interaction, information seeking, and self-documentation, contributing to enhanced communication, learning, and networking [3,4]. However, social media’s accessibility has led to social media addiction, negatively impacting mental and physical health, lifestyle, and social interactions [5]. Addiction, traditionally linked to substances, now includes behaviors like shopping, gambling, and media use [6]. Factors contributing to social media addiction include mental health issues, low self-esteem, peer pressure, and technological accessibility [7]. Mark Griffiths’ six criteria for addiction—salience, tolerance, mood modification, withdrawal, relapse, and conflict—apply to social media use [8]. Although not formally recognized in the International Classification of Diseases 11th Revision (ICD-11) or the 5th version of the Diagnostic and Statistical Manual of Mental Disorders (DSM-5), the Bergen Social Media Addiction Scale (BSMAS) helps identify potential addiction [9,10,11].

Research indicates various negative effects of social media, particularly among students. A 2021 review found that social media use correlates with lower academic engagement and grades, as well as with anxiety, depression, loneliness, and low self-esteem [7,12]. Physical health issues like tired eyes, headaches, and disrupted sleep patterns are common, along with neglecting meals and reduced exercise, leading to weight alterations [12].

### 1.2. Key Negative Impact

#### 1.2.1. Anxiety and Depression

Mental health, encompassing emotional, psychological, and social well-being, significantly affects students’ quality of life and academic engagement [13]. Numerous studies indicate a positive correlation between social media use and mental health issues like anxiety and depression among students from various countries, including Arab, Pakistani, French, Turkish, Chinese, Bengali, Indian, and Australian students [13,14,15,16,17,18,19,20,21,22]. Social media fosters comparisons and dissatisfaction, contributing to depression and anxiety, exacerbated by fear of missing out (FoMO) and low self-esteem [14,18]. The impact of social media use on mental health varies with the nature of use; communication-focused use is linked to higher depression among nursing students [19], while emotional investment is crucial in determining depression and anxiety risks [14].

#### 1.2.2. Sleep Disorders

Sleep is vital for mental and physical well-being. Internet addiction often correlates with poor sleep quality, pain, depression, and anxiety [23,24]. Excessive social media use is linked to increased sleep disorders among Turkish students [23]. However, Mahalingham et al. found no causal relationship between social media use and sleep quality after a week-long hiatus [25]. Lower melatonin levels due to overuse of mobile phones at night can disrupt sleep patterns [26].

#### 1.2.3. Pain and Headache

Smartphone use, especially for social media purposes, is associated with physical ailments like musculoskeletal pain. Nearly half of the participants among Ethiopian students with widespread smartphone use for social media reported neck pain [27]. Studies from Canada, Saudi Arabia, Malaysia, and India report a high prevalence of pain in the hand, shoulder, neck, and back among smartphone users [28,29,30,31]. Repetitive movements, poor posture, and prolonged use contribute to these issues, affecting quality of life and academic performance [32]. Screen time has been reported as a risk factor for headaches [33]. High levels of screen time exposure were found to be associated with migraine, but not with non-migraine headaches in young students of the Internet-based Students Health Research Enterprise (i-Share) project initiated by a couple of universities in France [33].

### 1.3. Use of Social Media and Consumption of Over-the-Counter (OTC) Medications

Non-pharmacological strategies like physical activity can alleviate conditions such as anxiety and depression, but this study focuses on the use of OTC medications linked to social media use among university students. A bibliometric study [34] from 2013 to 2022 primarily linked social media addiction with anxiety, depression, and extroversion, but not with the consumption of OTC medications. Our comprehensive literature search found no studies directly addressing this relationship, making our study exploratory.

OTC medications, unlike prescription-only medications, are available without a physician’s prescription and are intended for self-care. OTC medications’ accessibility allows individuals to manage mild ailments without a physician’s visit, easing the burden on healthcare services and allowing for a faster solution. For example, paracetamol and ibuprofen are used frequently for pain and are available as OTC medications in Norway. Statistics from the Norwegian Institute of Public Health (NIPH) show widespread use of OTC analgesics, with paracetamol and ibuprofen being top sellers [35]. Plant-based medicinal products are also available; for example, for stress relief or mild sleep issues (e.g., passionflower, valerian root, combined valerian root, hop flower, and lemon balm leaf, for stress relief and calming effects, and melatonin for sleep aid). Yet, overuse of these preparations can lead to adverse effects; for instance, there are concerns about the efficacy and safety of melatonin [36] and plant-based medicines [37].

Our study aimed to explore the potential correlation between social media use and several health outcomes among university students in Norway. Specifically, we sought to determine whether there is a correlation between social media use and anxiety, depression, sleep disorders, headaches, and musculoskeletal pain among university students. Additionally, we aimed to investigate the relationship between social media use and the consumption of OTC analgesics, stress relievers, and sleeping aids, as well as the frequency of their use among this population.

## 2. Materials and Methods

### 2.1. Research Design and Target Population

A quantitative research design using a cross-sectional study approach was selected. Data collection was conducted via a web-based questionnaire to gather quantifiable data.

The target group for this study was university students. According to Statistics Norway (ssb.no), Norway hosts approximately 300,000 students enrolled in universities or university colleges. Among these, about 63% are university students. The Ministry of Education and Research (regjeringen.no) oversees 21 educational institutions, with 10 of them classified as universities. The major universities include NTNU (Norwegian University of Science and Technology), UiO (University of Oslo), OsloMet (Oslo Metropolitan University), UiB (University of Bergen), UiT (the Arctic University of Norway), and USN (The University of South-Eastern Norway). The population for this study was defined as university students in Norway aged 18–29 pursuing bachelor’s, master’s, or professional studies, without diagnosed conditions and not using prescription medications.

### 2.2. Questionnaire

Previous studies utilized various scales and self-created questions to assess students’ social media use, anxiety, depression, sleep disturbances, and pain. The Bergen Social Media Addiction Scale (BSMAS), along with the Generalized Anxiety Disorder Scale (GAD-7), Patient Health Questionnaire (PHQ-9), and Bergen Insomnia Scale (BIS), were chosen for this study. These scales are freely accessible, publicly available, and have been translated into Norwegian. Moreover, the GAD-7, PHQ-9, and BIS are widely used in clinical practice, including among healthcare professionals. Table 1 provides details on the scoring and categorization for the GAD-7, PHQ-9, and BIS.

The GAD-7 (Generalized Anxiety Disorder Scale) assesses anxiety symptoms over the past two weeks, and it has been validated for use among university students [38,39,40]. The PHQ-9 (Patient Health Questionnaire) measures depressive symptoms over the past two weeks, and it has been widely validated for use [41,42,43,44]. The BIS (Bergen Insomnia Scale) evaluates sleep disturbances and fatigue over the past month, and it has been validated in student populations [45]. To assess the pain frequency, separate questions were formulated for headaches and musculoskeletal pain using a similar Likert scale to the GAD-7 and PHQ-9 to maintain consistency throughout the survey.

Given the absence of relevant studies on social media use and OTC medications consumption, specific questions regarding OTC medications were developed for this study.

#### 2.2.1. Questionnaire Structure

Initially, the questionnaire was structured using a Word document to outline all the questions and answer options. Subsequently, Nettskjema.no was employed to create the digital questionnaire for distribution. The questionnaire began with an introductory page detailing the project’s objectives, ensuring respondent anonymity, providing the estimated completion time, and establishing the criteria for participation. Additionally, it included a link to an information letter.

The questionnaire was divided into four parts, predominantly comprising closed-ended questions that were pre-coded and mandatory to answer. Some questions featured an “other” option, allowing participants to provide additional comments if desired.

Part 1 gathered descriptive data with seven closed-ended questions regarding gender, age, university affiliation, field of study, academic year, presence of diagnoses, and use of prescription medications. Participants answering affirmatively to the latter two questions were excluded from the study to ensure consistency in the data analysis. This section aimed to provide a demographic overview of the respondents.

Part 2 focused on students’ social media usage, encompassing three closed-ended questions. It explored the types of social media platforms used, average screen time spent on social media per day, and peak usage times. The BSMAS was included in this section to assess the degree of dependency or problematic use of social media among students. Part 2 aimed to characterize patterns of social media use and utilized the BSMAS scores in the subsequent statistical analyses.

Part 3 involved assessing symptoms related to anxiety, depression, sleep disturbances, headaches, and musculoskeletal pain. It employed validated scales: GAD-7, PHQ-9, and BIS for anxiety, depression, and sleep disorders, respectively. Additionally, two self-created questions were used to gauge the frequency of headaches and musculoskeletal pain. The results from this section were used to estimate the prevalence of these ailments and inform the statistical testing.

Part 4 focused on students’ utilization of OTC medications, comprising eight closed-ended questions. It collected data on the types of OTC analgesics and sedatives/sleeping aids used, purchasing sources, purposes of use, and students’ perceptions regarding the amount and frequency of use. The questions also explored students’ awareness of proper medication usage, including considerations regarding the amount and sources of information consulted for consumption guidance.

#### 2.2.2. Questionnaire Pilot Testing

Pilot testing is crucial to evaluate the feasibility and clarity of research instruments and protocols before implementation. The process aims to identify any ambiguities or complexities in the questionnaire and determine the necessary adjustments [46]. Two rounds of pilot testing were conducted with different groups, totaling ten individuals. Invitations to participate in the pilot testing were extended to recent university graduates, ensuring they closely resembled the target population for the study.

First Round of Pilot Testing:

The questionnaire was initially sent to a group of five individuals. They were instructed to assess the clarity, comprehensibility, and ease of answering the questions. Feedback was gathered through phone calls or messages. Based on their input, several adjustments were made:

In Part 2, the respondents found it challenging to accurately estimate their social media usage in hours using the original response options (“Less than 1 h”, “1–2 h”, “3–4 h”, “5–6 h”, “over 6 h”). To address this, the response categories were revised to “Less than 1 h”, “1–3 h”, “4–6 h”, and “Over 6 h”.

Part 3 originally included separate questions for headache frequency and musculoskeletal pain frequency. The respondents felt this redundancy was unnecessary, so the questions were combined into a single question each for headaches and musculoskeletal pain.

The initial requirement on the front page stating participants must not have a diagnosis or use prescription medications received negative feedback. To avoid any negative impressions, these requirements were removed from the front page and information letter. Instead, questions about diagnoses and prescription medication use were integrated into Part 1 of the questionnaire to facilitate the exclusion of ineligible respondents after the data collection.

Second Round of Pilot Testing:

A revised version of the questionnaire, incorporating changes based on the first round of pilot testing, was then administered to a second group of five individuals. The feedback received was minimal, confirming that most issues had been successfully addressed in the initial round.

Despite efforts to refine the questionnaire, some aspects remained unchanged due to constraints or established protocols. Some participants found the BIS challenging due to the variability in their sleep disturbances from week to week. However, since the majority of respondents found it manageable, the BIS was retained in its original form. Suggestions to alter the response options for the GAD-7 and PHQ-9, similar to the BSMAS, were not feasible. These scales are validated tools with established response formats critical for accurate symptom assessment and comparability across studies.

Overall, the pilot testing ensured the questionnaire was clear and feasible for the study’s target population, enabling adjustments that enhanced its usability and reliability.

#### 2.2.3. Distribution of Questionnaires and Data Collection

The questionnaire (available as Supplementary Material) was distributed primarily through Facebook and in one case through Instagram (Norwegian University of Life Sciences: NMBU) to reach university students in Norway. The student groups that were targeted on Facebook compromised the university students’ Facebook groups of NMBU, Nord University, NTNU, OsloMet, UiA (University of Agder), UiB, UiO, UiS (University of Stavanger), UiT, and USN.

A reminder was sent one week after the initial posting to groups that approved the questionnaire. The total membership of the Facebook groups where the questionnaire was published was approximately 50,000, which represents 26% of the total number of university students in Norway.

### 2.3. Ethical Considerations

Research ethics are paramount in safeguarding participants and ensuring robust research practices. In this study, extensive measures were taken to protect participants’ anonymity and ensure informed consent. Before the data collection commenced, the project was submitted to the Norwegian Centre for Research Data (NSD) for assessment by the Norwegian Agency for Shared Services in Education and Research (SIKT). The NSD determined that since the data collected would be completely anonymous and did not include identifiable health information, it did not require further review by the Data Protection Services (SIKT case number: 614179, dated 16 October 2023). Additionally, a submission was made to the Regional Committees for Medical and Health Research Ethics (REC), which confirmed that ethical approval was not necessary (REC case number: 691069, dated 13 November 2023). To uphold participant anonymity, the questionnaire was administered through Nettskjema.no with settings configured to collect anonymous responses without storing IP addresses, usernames, or other identifying information. Informed consent was obtained using a standardized information letter approved by the NSD. The letter outlined the purpose of the study and the participation requirements, and it provided contact information for the project owner for further inquiries. The wording of the questionnaire and information letter avoided terms like “addiction to social media” or “problematic use of social media” to mitigate potential negative reactions and encourage participation.

### 2.4. Data Processing and Statistical Tests

Raw data collected from the survey on Nettskjema.no were imported into Excel worksheets for initial processing, descriptive analysis, and statistical testing. This section details the methods used for data handling and the specific statistical tests conducted.

The questionnaire comprised 48 mandatory closed-ended questions and seven free-text fields, resulting in 55 variables. These variables were categorized into the categorical and continuous types, depending on their nature. For the categorical variables, the data presentation included frequency tables, bar charts, and mode calculations. The continuous variables were analyzed using measures of the central tendency (mean, median, mode) and dispersion (standard deviation, variance, quartiles).

Interpretation of the correlation coefficient was conducted with the aid of the available instruction [47], where values 0.00–0.199 are considered very weak; 0.20–0.399 weak; 0.40–0.599 medium; 0.60–0.799 strong; and 0.80–1.000 very strong.

To manage multiple comparisons and minimize the type 1 error, a Bonferroni correction was applied. With nine statistical tests conducted on the same dataset, the adjusted significance level was set at 0.05/9 = 0.006. Therefore, in the Spearman correlation analyses, a *p*-value below 0.006 indicated a statistically significant correlation.

All the statistical analyses were conducted using Excel’s Statistical Functions with the significance level set at 5% (0.05) and the adjusted value for multiple comparisons using Bonferroni correction.

## 3. Results

**Figure 1:**
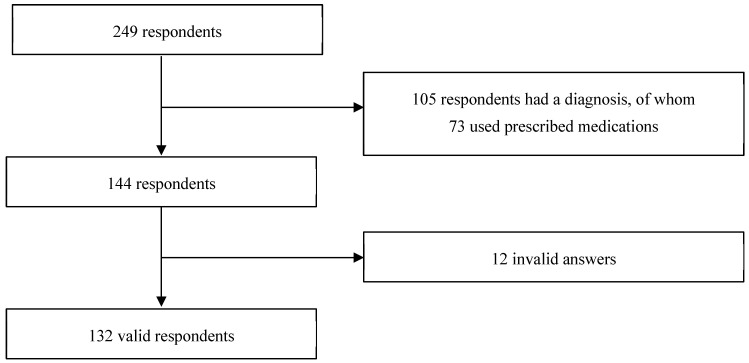
Flow diagram of the included responses for analysis.

The survey had 249 respondents. Among them, 105 indicated having one or more diagnoses, and out of these, 73 reported using prescription medications. Excluding 12 students whose responses were deemed irrelevant—2 due to using unspecified means and 10 due to irrelevant indications—132 students were included in the presentation and analysis Figure 1 shows a flow diagram of the included responses for analysis.

### 3.1. Presentation of the Findings

#### 3.1.1. Description of the Respondents’ Characteristics

The survey predominantly received responses from female students, constituting 80% (n = 106), with male students accounting for 20% (n = 26). The age group of 22–25 years was the most represented, while students aged 26–29 years provided the fewest responses. The largest number of respondents were affiliated with UiO (26%, n = 34), followed by UiA (18%, n = 24) and NMBU (17%, n = 23). Conversely, the smallest number of respondents came from USN (2%, n = 3). The responses were distributed relatively evenly among NTNU, UiT, UiB, and UiS, ranging from 4–6% (n = 5–8), and similarly for Nord University and OsloMet, which represented 8–10% (n = 10–13). The majority of respondents were first-, second-, or third-year students, with only one respondent in the sixth year of study. Approximately 30% (n = 38) of respondents were studying medicine and health sciences, while the remainder were pursuing other fields unrelated to health sciences (Table 2).

#### 3.1.2. Use of Social Media

**Figure 2:**
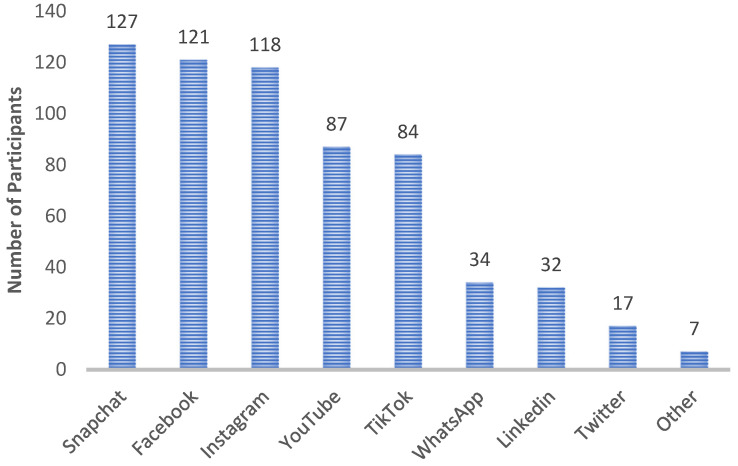
Overview of the social media platforms used by respondents. Respondents were instructed that they could select more than one platform.

All the respondents reported using more than one social media platform. Snapchat was the most popular, used by 127 students. Twitter had the fewest users, totaling 17. Figure 2 illustrates the predominant social media platforms used by the respondents, namely Snapchat, Facebook, and Instagram. Additionally, seven students indicated using other social media platforms not listed in the questionnaire, such as Telegram, Reddit, Tumblr, and BeReal.

The majority of respondents, 75% (n = 99), used social media in the evenings, while 25% (n = 33) used it during the day. The usage patterns varied, with 48% (n = 63) spending 1–3 h and 46% (n = 61) spending 4–6 h daily on social media. Only 2% (n = 3) used social media for less than 1 h, and 4% (n = 5) used it for more than 6 h daily.

Regarding social media addiction, as measured by the BSMAS scores, only 5% (n = 7) of students could be classified as addicted. The remaining 95% (n = 125) had scores indicating non-addictive behavior, with a total score of less than 24 (Table 3). The average BSMAS score was 16, indicating a relatively high level of social media use among the respondents.

#### 3.1.3. Anxiety, Depression, Sleep Disorders, and Pain

Approximately half of the respondents experienced mild symptoms of anxiety and depression. Specifically, 8% (n = 10) reported severe anxiety, while 15% (n = 20) experienced fairly severe or severe depression. About 24% (n = 32) had no symptoms of anxiety, and 23% (n = 30) reported no symptoms of depression.

Concerning sleep disorders, 64% (n = 85) scored at least 6 on the BIS, indicating the likelihood of having a sleep disorder.

**Figure 3:**
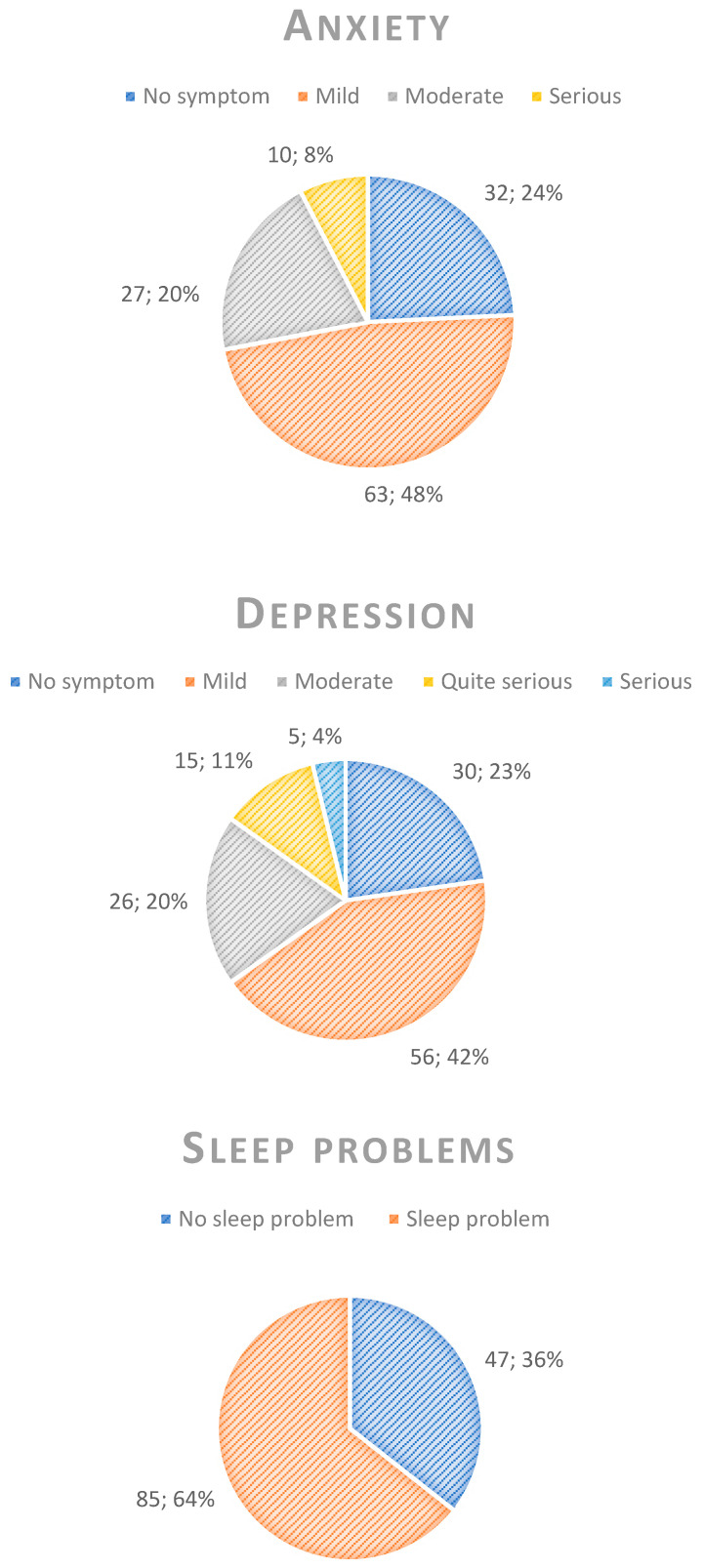

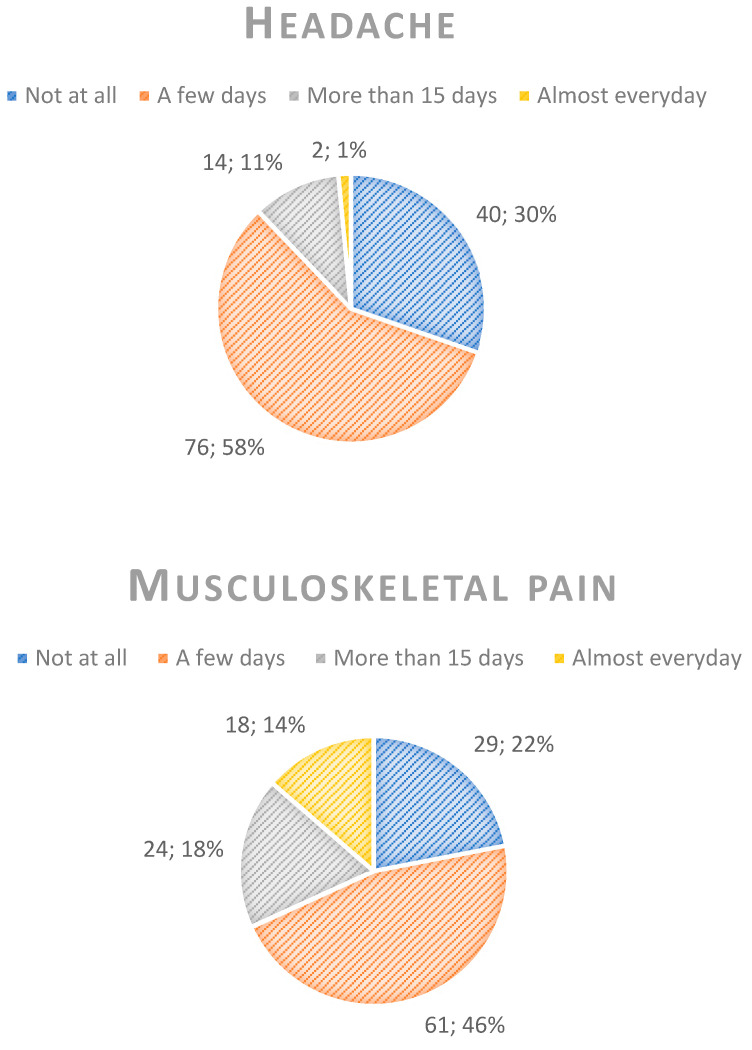
Response distribution among the respondents (n = 132) related to anxiety symptoms, depression symptoms, sleep disturbances, headache, and musculoskeletal pain. Please note that the numbers in the pie charts indicate the number of participants and the corresponding percentage, separated with semicolons.

In terms of their physical health, 58% (n = 76) of respondents reported experiencing headaches for a few days in the past month, while 46% (n = 61) reported musculoskeletal pain during the same period. Fewer respondents experienced headaches or musculoskeletal pain almost every day, with 30% (n = 40) not experiencing headaches and 20% (n = 29) not experiencing musculoskeletal pain at all. Graphical representations of these findings are presented in pie charts, detailing the prevalence of each negative effect assessed (Figure 3).

#### 3.1.4. Use of OTC Medications

Of the 132 respondents, 56% (n = 74) had used OTC analgesics, sedatives, sleeping aids, or a combination of these in the past month. Specifically, 64 students used medications containing paracetamol, and 28 students used non-steroidal anti-inflammatory drugs (NSAIDs) such as ibuprofen, naproxen, and diclofenac gel. Additionally, 18 students used sleeping aids containing melatonin. Three used aspirin, three Sedix (contains passion flower extract, which is traditionally used to relieve mild symptoms of anxiety, such as nervousness, worries or irritability), two Lunixen (a plant-based medicine that contains dried extract of valerian root and is used against restless sleep, too early awakening and for improving the sleep rhythm, as well as for relieving mild symptoms of restlessness), two Valerina Forte (a plant-based medicine used to relieve mild restlessness and sleep disorders that contains extract of valerian root), two Valerina Natt (a traditional plant-based medicine for use in mild sleep disorders), one Fenazon-Koffein (used for light and moderate pain, e.g., for headaches, toothaches and menstrual pains), and one Pascoflair (a traditional plant-based medicine used to relieve mild symptoms of restlessness and to facilitate falling asleep). Respondents could select more than one option. Among the 74 respondents, 16 had used at least one OTC analgesic and one sedative or sleeping aid.

A total of 53 students purchased OTC medications from pharmacies, 38 from grocery stores, and 1 from a health food store. Additionally, four students used an online pharmacy, two ordered from an online store, and two received medicines from their families.

Only 16% (n = 12) indicated that they were not concerned with the correct amount of OTC analgesics and/or sedatives/sleeping aids, whereas 84% (n = 62) expressed concern about the proper amount. More than half of the students, 57% (n = 42), reported occasional use of these medications in the past month, while only 7% (n = 5) used them daily (Table 4).

The majority of students used multiple sources to obtain information about consumption (amount/frequency). The most commonly used source was the packs/package inserts, as utilized by 48 students. Additionally, 35 students relied on their own knowledge, and 24 received help from a pharmacy employee. The internet was used by 18 students, while 16 consulted acquaintances. Three students selected “other”, with two specifying “doctor” and “own impulses” as their sources of information.

**Figure 4:**
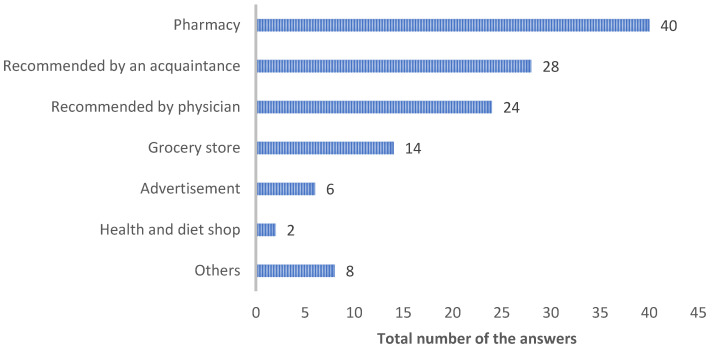
Overview of how the respondents became aware of over-the-counter analgesics and sedatives/sleeping aids. Respondents could select more than one option if needed.

A total of 40 students, representing more than half of those who used OTC medications, became aware of these medications through various sources, with pharmacies being the most popular (Figure 4). Additionally, eight students selected “others” as their source of awareness. Four of these students cited their parents, while the remaining four believed they had always known about OTC medications.

**Figure 5:**
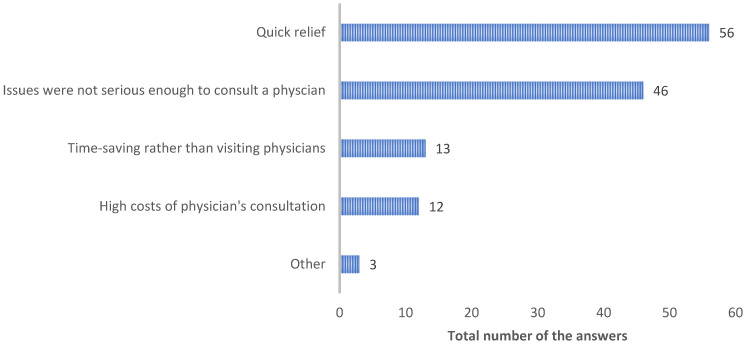
Distribution of respondents’ reasons for choosing to buy over-the-counter medications. Respondents were asked to select more than one option if needed.

Most respondents indicated that they purchased OTC medications because they felt their ailments were not severe enough to warrant a doctor’s visit or because they needed quick relief (Figure 5). All the students who selected time-saving as a factor also selected both quick relief and the perceived severity of their ailment. Additionally, 5 out of 74 students checked all four factors. Three students selected “others”, with two mentioning exhaustion due to a hectic study life.

### 3.2. Statistical Tests

Statistical tests were carried out to investigate the association between the use of social media and negative effects, and the association between the use of social media and the consumption of OTC analgesics and sedatives/sleeping aids.

#### 3.2.1. Use of Social Media and Potential Negative Effects

Respondents who had no or mild symptoms of anxiety and depression, no sleep disturbances, and no pain (headache and musculoskeletal pain) were excluded from the statistical tests. This focus ensured that the analysis targeted respondents significantly affected by these negative effects.

Anxiety

The correlation coefficient between social media use and anxiety was 0.09 and the *p*-value was 0.60, larger than the adjusted significance level (0.006), presenting no correlation.

Depression

The correlation coefficient between social media use and depression was 0.04, with a *p*-value of 0.79, which indicated no correlation.

Sleep Disorders

The correlation coefficient between social media use and sleep disturbances was 0.21, with a *p*-value of 0.05, which indicated no significant correlation.

Pain

For headaches, the correlation coefficient was 0.24, and for musculoskeletal pain, it was 0.20. The *p*-values were 0.02 for headaches and 0.04 for musculoskeletal pain. Both *p*-values were greater than the adjusted significance level (0.006), and therefore, no correlation became evident.

#### 3.2.2. Use of Social Media and Consumption of Over-the-Counter Medications

Use of OTC Medications

Logistic regression analysis for both OTC analgesics and sedatives/sleeping aids showed larger *p*-values (0.15 and 0.17, respectively) than the adjusted significance level (0.006). This indicates that no association can be found between the use of social media and the consumption of OTC medications.

Frequency of usage of OTC medications

Respondents who answered “occasionally” were excluded from the statistical tests examining the correlation between social media use and the frequency of consumption of OTC medications. The term “occasionally” does not specify how often students use OTC medications.

Analgesics: The correlation coefficient for the frequency of use of analgesics was 0.17 and the *p*-value was 0.09. This indicated that no correlation existed between the use of social media and the frequency of consumption of OTC analgesics.

Sedatives/sleeping aids: The correlation coefficient for the frequency of use of OTC sedatives/sleeping aids was 0.09, with a *p*-value of 0.42. This indicated that no correlation existed between social media use and the frequency of use of these medications.

## 4. Discussion

### 4.1. Discussion of Methods

#### 4.1.1. Literature Search

The methodological approach of this study was twofold: a comprehensive narrative literature [48] and a quantitative survey. The literature search was conducted to identify the state of the art within the relationship between social media use and potential negative effects such as anxiety, depression, sleep disturbances, and pain, and also to identify whether the current literature has presented any potential association between social media use and consumption of over-the-counter medications. The findings from the literature search provided essential insights into existing research gaps, guided the formulation of the research questions, informed the construction of the questionnaire, and facilitated the interpretation of our results.

Narrative reviews, unlike systematic reviews, do not adhere to a formal protocol but provide a comprehensive overview of the existing literature. To ensure transparency in the search process, the PICO framework (specifies the type of patient or population, type of interventions and comparisons if there are any, and the type of outcome) was utilized to identify relevant keywords, which guided the actual search and the selection of pertinent articles. This process might have introduced biases, defined as systematic errors that distort study findings. Selection bias is a concern, as relevant studies may have been overlooked due to search constraints. The language was restricted to Norwegian and English, and searches for negative effects were limited to publications between 2018 and 2023. Moreover, the susceptibility of findings to publication bias is acknowledged, wherein studies favoring a positive association between social media use and negative effects might have influenced the availability of findings on this topic. Hence, a cautious interpretation of the literature review’s findings is warranted.

#### 4.1.2. Research Design

The quantitative approach adopted in this study allowed the responses to be quantified, facilitating the application of relevant statistical tests to establish any potential relationships. A cross-sectional study design with the aid of questionnaires offers a snapshot of reality, assuming representativeness within the population, and is advantageous for efficiently gathering large volumes of data in a short time frame. However, cross-sectional studies, such as the one employed here, carry limitations. They capture data at a single point in time, providing only correlational insights (if any) rather than establishing causation because all the variables are measured concurrently. To ascertain causality, longitudinal or experimental designs are necessary. An observational longitudinal study, spanning from adolescence to early adulthood, could explore how social media exposure influences the development of mental disorders, sleep disturbances, pain, and eventually the consumption of OTC medications, or changes therein.

#### 4.1.3. Population

The study population was confined to university students, with a focus on university rather than university college students since a higher proportion of students are enrolled in universities in Norway. The age and educational level were further delimited to specify the population under study. Previous research highlights extensive studies on young adults, with Norway defining “young” as individuals aged 18–25. According to the Database for Statistics on Higher Education (DBH), the majority of Norwegian university students fall within the 20–29 age group. Thus, the age range for this study was set from 18 to 29 years. This study focused on Norwegian bachelor’s, master’s, and professional programs, which are the most prevalent at universities. A bachelor’s program typically spans 3 years, a master’s program 2 or 5 years, and professional studies 5 or 6 years. However, some limitations, such as specific study years, may have excluded potentially relevant participants. Adjusting the survey to inquire about the type of study program rather than specific years might have yielded better insights, for example, the health science field including psychology, nursing, medicine, and pharmacy. Future research priorities can highlight educational fields and levels.

Making the distinction of study fields based on the standard divisions of natural, technical, humanities, and social sciences would be optimal.

Confounding variables, which correlate with both the dependent and independent variables under the study, were considered. Neglecting these variables can lead to spurious correlations and misinterpretation of the findings [49]. Diagnoses and the use of prescription medications are potential confounders. Diagnoses may diminish life satisfaction and foster social isolation, increasing susceptibility to mental health issues and pain. Consequently, individuals may resort to OTC medications like analgesics or sedatives/sleeping aids. Similarly, the consumption of prescription medication, which can induce side effects like insomnia or mood changes, may prompt increased social media use to mitigate isolation and boost well-being. Consequently, the use of OTC medications could be more related to other health conditions irrelevant to social media use. To mitigate these complexities, respondents with diagnoses and/or using prescribed medications were excluded from the analysis to avoid misinterpretation of the findings. In addition, considering recall bias, we cannot ignore the possibility of the consumption of OTC medications by the participants for other health issues irrelevant to social media use. Another confounding factor is related to the familiarity or unfamiliarity of the participants with medications and their indications. For instance, paracetamol, which is known as an OTC analgesic, has been reported to be used to reduce anxiety and stress. It might partially be due to comorbid conditions where pain and psychological problems coexist. A recent study in Norway reported that sex, pain, anxiety, and depression were interrelated and could act as strong predictors of frequent OTC analgesics use [50]. This study recommended that consumption of OTC analgesics among youth, particularly females, with anxiety and depression should be cautious and monitored. Attempts are ongoing to develop strategies to promote the responsible use of OTC analgesics, and in this domain, the potential role of community pharmacists can be remarkable [51,52].

#### 4.1.4. Distribution of Questionnaires and Data Collection

The survey’s response rate was notably low, with only 249 participants out of approximately 188,000 university students in Norway. Several factors contributed to this reduced reach. Firstly, the questionnaire could not be posted on the official Facebook pages of nine out of ten universities. Instead, it had to be shared in unofficial student-created Facebook groups, which typically have fewer members than official university pages. Moreover, posts in these groups required approval from group administrators to ensure relevance to their members, which further limited the visibility and response rates, especially across multiple universities. Secondly, Facebook groups were unavailable for some university campuses of UiT, NTNU, USN, and Nord University, resulting in unequal participation rates among universities. Thirdly, the survey was distributed in November, coinciding with exam periods for many students. This likely reduced the social media activity and engagement, thereby affecting the visibility of the survey posts. Fourthly, students who were not members of these Facebook groups were unlikely to see or participate in the online survey distributed via these channels. Additionally, inactive or former students may have comprised a portion of group memberships, further reducing the potential respondents. To improve the respondent numbers, an alternative distribution method could involve placing posters with QR codes on university campuses. This approach would ensure accessibility to all students, irrespective of their membership of Facebook groups. However, students who do not frequently visit campus might miss the posters. Using various distribution channels, direct posts, and wide visibility on campuses in terms of both digital posters and traditional posters in common environments, such as libraries and eating areas, dormitories, etc., are encouraged for future studies. Overall, practical considerations and careful strategic plans in survey distribution methods are highly important and must be taken into account for future studies of this kind.

#### 4.1.5. Other Methodological Considerations

In this study, “use of social media” was designated as the independent variable to investigate its potential relationships with anxiety, depression, sleep disturbances, pain, and the consumption of OTC medications. Multiple regression could have explored whether anxiety, depression, sleep disturbances, and pain impact social media use and potentially influence the consumption of OTC medications. The small sample size prevented meeting the criteria for this analysis. Hence, bivariate analyses, Spearman correlation, and logistic regression were used to test the study hypotheses. A larger sample size is recommended for the next investigation.

The anonymity of the questionnaire promoted reliability by encouraging honest responses, especially for sensitive topics like mental health and sleep disorders [53]. However, relying on self-reported surveys can introduce memory bias, potentially reducing the reliability. Limitations in the response options for questions on pain frequency and the consumption of OTC medications might have compromised the study validity. Ambiguities in response interpretations, such as varying definitions of terms like “occasionally,” could also have skewed the results. Improved response specificity could mitigate such issues in future studies.

### 4.2. Discussion of Results

#### 4.2.1. Use of Social Media and Potential Negative Effects

Anxiety and Depression:

The analysis revealed that a considerable portion of respondents experience moderate to severe anxiety (28%) and depression symptoms (35%). Despite this prevalence, the Spearman correlation analyses between social media use and these negative effects yielded high *p*-values, suggesting a lack of association. This finding contrasts with the existing literature, which often reports a positive correlation between social media use and mental health issues among students across various countries [13,14,15,16,17,18,19,20,21,22]. The discrepancy in findings regarding the correlation between social media use and anxiety/depression in this study could be attributed to the limitations of the BSMAS scale. The BSMAS primarily assesses addiction to social media without accounting for the actual time spent on these platforms. Many respondents in this study exhibited low BSMAS scores despite spending significant hours on social media and experiencing moderate to severe anxiety and/or depression symptoms. This suggests that relying solely on the BSMAS may not adequately capture the nuances of social media usage in relation to mental health outcomes. This observation aligns with studies emphasizing the importance of considering actual screen time rather than just addiction scales like the BSMAS. Research has shown, for example, that children spending extended periods on smartphones daily are more likely to exhibit depressive symptoms [54].

Factors contributing to depression among university students include low self-esteem, academic pressures, stress, financial challenges, and biological factors such as hormonal changes and genetics [13,55]. For instance, studies indicate that students in specific academic programs or living arrangements may experience varying levels of depression. Pharmacy students with problematic social media use, for example, reported lower rates of depression compared to students in other disciplines [55]. Similarly, sports students facing career concerns and rigorous training schedules may also experience heightened depression symptoms [16].

It is important to state that the inclusion of both negative and positive impacts of social media exposure on youth mental health would be beneficial, as there has been more emphasis on the harmful impacts of social media on mental health [56,57]. Recent qualitative study results [58] highlight opportunities for positive influences, personal expression, and social support, contributing to positive mental health among youth, which has also been reviewed [59].

#### 4.2.2. Sleep Disorders

The correlation analysis indicated a trend toward a slight positive correlation between social media use and sleep disturbances. However, this was not significant. We could not rule out whether higher scores on the BSMAS are aligned with higher scores on the BIS. Previous studies have found a positive association between social media use and sleep disorders [23,24,26]. Despite our negative finding, over 64% of respondents reported being affected by sleep disturbances based on their BIS scores. Perhaps factors other than social media use contribute to sleep disturbances among university students. One possible contributing factor highlighted in the literature is gender, with female students typically experiencing more sleep disturbances compared to male students [60]. In this study, there was an overrepresentation of female respondents, which may have influenced the high prevalence of sleep disturbances observed. Other factors contributing to sleep disorders among university students include illness, anxiety, academic stress, negative thoughts, and other late-night activities [60].

#### 4.2.3. Pain (Headache and Musculoskeletal Pain)

The correlation analysis presented a trend toward a slight positive correlation between social media use and both headache and musculoskeletal pain. However, this was not significant. Therefore, we cannot conclude that students who tend to use social media more intensively may also experience headaches and musculoskeletal pain more frequently compared to those who do not exhibit problematic social media use behaviors. Previous research examining smartphone use has found a higher prevalence of pain among frequent users [27,28,29,30,31,32]. Our survey revealed that a significant proportion of respondents reported experiencing headaches (71%) and musculoskeletal pain (78%). Other factors beyond social media usage likely play a substantial role in the prevalence of pain among the students who participated in our study. Among medical students, for example, the demanding nature of their studies has been linked to higher rates of headaches. The stress associated with learning, exam pressures, and the need for sustained concentration throughout their studies might contribute to this phenomenon [31]. Additionally, factors such as financial stress, quality of life, and personal strains have been associated with headaches and musculoskeletal pain [27].

Taken together these findings show that social media use alone may not fully explain the high prevalence of sleep disturbances and pain reported by university students, at least not among our study participants. Future research should explore additional contributing factors to provide a more collective understanding of their impact on students’ well-being.

#### 4.2.4. Use of Social Media and Consumption of OTC Medications

The statistical tests conducted in this study did not find significant correlations between the use of social media and the consumption of OTC medications. This suggests that social media use alone may not directly influence how university students utilize OTC medications. Despite the lack of statistical correlation, the survey results indicated that analgesics were the most commonly used OTC medications among the respondents, with paracetamol and NSAIDs being the most popular choices. This aligns with national statistics showing paracetamol and ibuprofen to be the top-selling OTC pain relievers in Norway [35]. In contrast, sedatives/sleeping aids, for example, melatonin, were less frequently used compared to analgesics among the respondents. This usage pattern reflects broader trends where melatonin is commonly used by young people but remains less prevalent compared to pain relievers.

The majority of students reported purchasing OTC medications from community pharmacies, indicating a preference for reliable sources despite the availability in other venues like supermarkets and online stores. However, fewer students sought advice on the amount from pharmacy employees, suggesting a potential gap in accessing professional guidance. Those students who indicated using OTC medications occasionally expressed concerns about the correct amount or frequency, indicating a cautious approach toward their use. Many students acquired knowledge about OTC medications from sources like package inserts, the internet, personal knowledge, and acquaintances, demonstrating varied levels and reliability of acquired information. We identified that the decision to use OTC medications among students was primarily influenced by the need for rapid relief and the severity of symptoms, often bypassing medical consultations. This self-care approach highlights convenience and cost-saving benefits but also warns of the importance of pharmacist-initiated discussions to ensure safe and effective usage of OTC medications picked by young customers. Community pharmacists can play a critical role as students’ primary source of information on OTC medications, emphasizing their responsibility to provide accurate usage guidance and assess the appropriateness of the choice of the product based on the symptoms described by the client or the possibility of referral for other non-pharmacological strategies [51,52].

While this study did not establish a direct link between social media use and the consumption of OTC medications, it highlighted significant patterns in medication preferences, sources of information, and decision-making factors among university students. Future studies could explore additional contextual factors influencing students’ choices and behaviors regarding OTC medications to enhance public health interventions and optimal community pharmacy services.

### 4.3. Study Limitations, Potential Implications, and Future Directions

Further research is encouraged. Conducting similar types of studies with a larger sample size could help validate the findings and provide a deeper understanding. Future research avenues include longitudinal studies to explore changes over time in social media usage patterns and OTC medications behaviors among university students. Qualitative methods such as focus group interviews could also provide deeper insights for understanding the relationship between social media and the use of OTC medications among university students while adjusting for various elements, e.g., sex, gender, study type, and level. We faced several limitations such as a small sample size that contributed to low power for proper statistical analysis. The skewness in the respondents’ distribution among various demographic factors can potentially influence the overall findings. Based on the findings from this study, we could not present a significant association between the tested parameters and social media use. However, our findings are still valuable, indicating a trend where a substantial number of students who were asked about the use of social media reported experiencing health conditions and consuming OTC medications for quick symptom relief. These insights can inform measures aimed at enhancing health literacy among university students about social media and the consumption of OTC medications. Educational efforts could focus on fostering responsible social media habits and promoting health awareness. Student organizations could expand their services to include educational resources or counseling facilities, addressing the healthy use of social media and the importance of healthy lifestyle choices among university students. Incorporating study curriculum components that educate university students about the effects of social media on mental health, sleep quality, and pain can empower them to make informed decisions and reduce negative health outcomes. Additionally, enhancing the mental health support services or counseling possibilities available onsite at university campuses can be beneficial.

Community pharmacists can play a crucial role as the primary providers of OTC medications. Pharmacists must engage with young clients purchasing OTC medications more proactively [61,62]. Initiating conversations about the reasons behind their choice of medications can provide valuable insights and help determine appropriate choices for usage or consider a consultation for alternative choices, including social prescription, referrals, and non-pharmacological strategies [63]. Pharmacists can also contribute to the expansion of university students’ health literacy by offering educational sessions at universities. Topics could include proper OTC medications usage, potential side effects, drug interactions, and the importance of consulting healthcare providers. Utilizing social media platforms, which are popular among young adults, to disseminate evidence-based information on safe medication practices could further enhance awareness and responsible prescription-free medication use.

## 5. Conclusions

This study did not find any significant correlation between social media use and pain or sleep disturbances, nor between social media use and the consumption of OTC medications, among university students in Norway. We only observed slight positive trends and hence interpreted the results cautiously in light of the study’s limitations, including the small sample size, suboptimal distribution timing and channels for the online questionnaire, and the presence of confounding factors. The important finding is, however, that a pattern was noticeable among university students who self-reported experiences of anxiety, depression, sleep disturbances, and pain, with half of the participants consuming OTC medications. In this line, student organizations, educational entities, and community pharmacists can serve as pivotal resources to contribute to guiding, counseling, and providing potential preventive measures and strategies. Future research with a focus on this theme is encouraged to provide evidence-based information on social media use and its potential negative effects, including patterns of OTC consumption among young adults.

## Figures and Tables

**Table 1:** Overview of the GAD-7 (59), PHQ-9 (60), and BIS (61).

Mapping Tools	Number of Questions	Scoring	Categorization
GAD-7	7	5–9	Mild anxiety
		10–14	Moderate anxiety
		15–21	Severe anxiety
PHQ-9	9	5–9	Mild depression
		10–14	Moderate depression
		15–19	Quite severe depression
		20–27	Severe depression
BIS	6	≥3 on at least one of the first three questions and ≥3 on at least one of the last two questions.	Sleep disorder

**Table 2:** Respondents’ distribution of responses by gender, age, year of study, and university (n = 132).

Variable	Value	Number	Percent (%)
Gender (n = 132)	Man	26	20%
	Woman	106	80%
Age (n = 132)	18–21 years	47	36%
	22–25 years	76	57%
	26–29 years	9	7%
Field (n = 132)	Medicine and health Sciences	38	29%
	Other	94	71%
Academic year (n = 132)	1 year	33	25%
	2 years	30	23%
	3 years	35	27%
	4 years	16	12%
	5 years	17	13%
	6 years	1	1%
University (n = 132)	NMBU	23	17%
	Nord University	13	10%
	NTNU	8	6%
	OsloMet	10	8%
	UiT	6	5%
	UiA	24	18%
	UiB	5	4%
	UiO	34	26%
	UiS	6	5%
	USN	3	2%

**Table 3:** Respondents’ response distribution by the time spent, daily use, and BSMAS score (n = 132).

Variable	Value	Number	Percent (%)
Time spent (n = 132)	<1 h	3	2%
	1–3 h	63	48%
	4–6 h	61	46%
	>6 h	5	4%
Daily use (n = 132)	Daytime	33	25%
	Evenings	99	75%
BSMAS (n = 132)	Dependent (addicted)	7	5%
	Not dependent (not addicted)	125	95%

**Table 4:** Respondents’ distribution of responses to questions related to the consumption frequency of over-the-counter (OTC) medications (n = 74).

Variable	Value	Number	Percent (%)
Concerned about the right amount (n = 74)	Yes	62	84%
	No	12	16%
Frequency of over-the-counter medications consumption (n = 74)	Daily basis	5	7%
	Weekly basis	13	17%
	Monthly basis	14	19%
	Occasionally	42	57%

## Data Availability

Original data are available.

## Supplementary Materials

The following supporting information can be downloaded at https://www.mdpi.com/article/10.3390/ejihpe14080147/s1, The original Norwegian questionnaire.
