# Supplementary material for: Social Media Use and Consumption of Prescription-Free Medications for Anxiety, Sleep, and Pain among Norwegian University Students
Source: Eur J Investig Health Psychol Educ. 2024 Aug 1;14(8):2205–25. doi: 10.3390/ejihpe14080147 (PMC11353468; doi:10.3390/ejihpe14080147)
Supplement: Supplementary file 1 [file ejihpe-14-00147-s001.zip › ejihpe-3105804-supplementary.pdf]

Nettskjema

Sammenheng mellom bruk av sosiale medier og reseptfrie midler blant studenter i Norge

Forskning har vist at symptomer på hyppig bruk av sosiale medier kan inkludere depresjon, angst, søvnforstyrrelser og smerter. Formålet med masterprosjektet er å kartlegge om det er en korrelasjon mellom bruk av sosiale medier og forbruk av reseptfrie beroligende midler, sovemidler og/eller smertestillende blant universitetsstudenter i Norge.
Spørreskjema består av fire deler: 1)bakgrunnsopplysninger, 2)ditt forhold til sosiale medier, 3)kartlegging av diverse symptomer og 4)din bruk av reseptfrie midler. Personopplysninger skal ikke bli samlet og alle svar er helt anonyme. Det vil ta deg 5-10 min å fullføre undersøkelsen.
Kritererier for å delta i spørreundersøkelsen:
1. Du er mellom 18 og 29 år.
2. Du studerer bachelor- eller masterstudiet ved et universitet i Norge.

Du kan også laste ned et detaljert informasjonsskriv med kontaktopplysninger her: Informasjonsskriv
Ved å trykke neste gir du samtykke til å delta.

1) Bakgrunnsopplysninger

Kjønn

- Mann
- Kvinne
- Annet

Alder

- 18-21
- 22-25
- 26-29

Universitet

- NMBU
- Nord Universitet
- NTNU
- OsloMet
- UiT Norges arktiske universitet
- Universitetet i Agder
- Universitetet i Bergen
- Universitetet i Oslo
- Universitetet i Stavanger
- Universitetet i Sørøst-Norge

Fagområde

- Medisin- og helsefag
- Annet

Studieår

- 1.år

- 2.år
- 3.år
- 4.år
- 5.år
- 6.år

Har du en eller flere diagnoser som er stilt av en kliniker? (eks. diabetes, epilepsi, migrene, psykisk lidelse, smerter)

- Ja
- Nei

Bruker du reseptbelagte medisiner for å behandle/forebygge din diagnose?

Dette elementet vises kun dersom alternativet «Ja» er valgt i spørsmålet «Har du en eller flere diagnoser som er stilt av en kliniker? (eks. diabetes, epilepsi, migrene, psykisk lidelse, smerter)»

- Ja
- Nei

2) Ditt forhold til sosiale medier

Velg sosiale medier som du bruker.

Du kan velge flere alternativer.

- Facebook
- Instagram
- LinkedIn
- Snapchat
- TikTok
- Twitter
- WhatsApp
- YouTube
- Andre
- Jeg bruker ikke sosiale medier.

Hvis andre, vennligst oppgi her.

Dette elementet vises kun dersom alternativet «Andre» er valgt i spørsmålet «Velg sosiale medier som du bruker.»

Anslå det gjennomsnittlige antall timer du bruker på sosiale medier per dag.

Dette elementet vises kun dersom alternativet «Facebook eller Snapchat eller Instagram eller TikTok eller WhatsApp eller Twitter eller YouTube eller LinkedIn eller Andre» er valgt i spørsmålet «Velg sosiale medier som du bruker.»

- Under 1 time
- 1-3 timer
- 4-6 timer
- Over 6 timer

Når på døgnet bruker du sosiale medier mest?

Dette elementet vises kun dersom alternativet «Facebook eller Snapchat eller Instagram eller TikTok eller WhatsApp eller Twitter eller YouTube eller LinkedIn eller Andre» er valgt i spørsmålet «Velg sosiale medier som du bruker.»

- Dagstid
- Kveldstid

Nettskjema

Nedenfor finner du **seks spørsmål** som er knyttet til **din bruk av sosiale medier**. Velg svaralternativet som beskriver deg best.

Snu mobilskjermen i landskapsmodus for å få best visning.

Hvor ofte i løpet av det siste året har du...

Brukt mye tid til å tenke på sosiale medier eller planlegge bruk av sosiale medier

Dette elementet vises kun dersom alternativet «Facebook eller Snapchat eller Instagram eller TikTok eller WhatsApp eller Twitter eller YouTube eller LinkedIn eller Andre» er valgt i spørsmålet «Velg sosiale medier som du bruker.»

Svært sjelden

Sjelden

Av og til

Ofte

Svært ofte

Følt en trang til å bruke sosiale medier mer og mer

Dette elementet vises kun dersom alternativet «Facebook eller Snapchat eller Instagram eller TikTok eller WhatsApp eller Twitter eller YouTube eller LinkedIn eller Andre» er valgt i spørsmålet «Velg sosiale medier som du bruker.»

Svært sjelden

Sjelden

Av og til

Ofte

Svært ofte

Brukt sosiale medier for å glemme personlige problemer

Dette elementet vises kun dersom alternativet «Facebook eller Snapchat eller Instagram eller TikTok eller WhatsApp eller Twitter eller YouTube eller LinkedIn eller Andre» er valgt i spørsmålet «Velg sosiale medier som du bruker.»

Svært sjelden

Sjelden

Av og til

Ofte

Svært ofte

Prøvd å kutte ned på bruken av sosiale medier uten å lykkes

Dette elementet vises kun dersom alternativet «Facebook eller Snapchat eller Instagram eller TikTok eller WhatsApp eller Twitter eller YouTube eller LinkedIn eller Andre» er valgt i spørsmålet «Velg sosiale medier som du bruker.»

Svært sjelden

Sjelden

Av og til

Ofte

Svært ofte

Blitt rastløs eller urolig dersom du har blitt forhindret fra å bruke sosiale medier

Dette elementet vises kun dersom alternativet «Facebook eller Snapchat eller Instagram eller TikTok eller WhatsApp eller Twitter eller YouTube eller LinkedIn eller Andre» er valgt i spørsmålet «Velg sosiale medier som du bruker.»

Svært sjelden

Sjelden

Av og til

Ofte

Svært ofte

Brukt sosiale medier så mye at det har gått utover jobben og/eller studier

Dette elementet vises kun dersom alternativet «Facebook eller Snapchat eller Instagram eller TikTok eller WhatsApp eller Twitter eller YouTube eller LinkedIn eller Andre» er valgt i spørsmålet «Velg sosiale medier som du bruker.»

Svært sjelden

Sjelden

Av og til

Ofte

Svært ofte

3) Kartlegging av diverse symptomer

Denne delen består av fire deler.
Snu mobilskjermen i landskapsmodus for å få best visning.

Del 1 - Nedenfor finner du **sju spørsmål** knyttet til **angst**. Velg svaralternativet som beskriver deg best.

Hvor ofte har du følt på disse symptomene i løpet av den siste måneden?

Følt deg nervøs, engstelig eller veldig stresset

Ikke i det hele tatt

Noen dager

Mer enn halvparten av dagene

Nesten hver dag

Ikke klart å stoppe eller kontrollere bekymringene dine

Ikke i det hele tatt

Noen dager

Mer enn halvparten av dagene

Nesten hver dag

Bekymret deg for mye om ulike ting

Ikke i det hele tatt

Noen dager

Mer enn halvparten av dagene

Nesten hver dag

Hatt vansker med å slappe av

Ikke i det hele tatt

Noen dager

Mer enn halvparten av dagene

Nesten hver dag

Vært så rastløs at det har vært vanskelig å sitte stille

Ikke i det hele tatt

Noen dager

Mer enn halvparten av dagene

Nesten hver dag

Blitt lett sint eller irritert

Ikke i det hele tatt

Noen dager

Mer enn halvparten av dagene

Nesten hver dag

Følt deg redd som om noe forferdelig kunne komme til å skje

Ikke i det hele tatt

Noen dager

Mer enn halvparten av dagene

Nesten hver dag

Del 2 - Nedenfor finner du **ni spørsmål** knyttet til **depresjon**. Velg svaralternativet som beskriver deg best.

Hvor ofte har du følt på disse symptomene i løpet av den siste måneden?

Lite interesse for eller glede over å gjøre ting

Ikke i det hele tatt

Noen dager

Mer enn halvparten av dagene

Nesten hver dag

Følt deg nedfor, deprimert eller fylt av håpløshet

Ikke i det hele tatt

Noen dager

Mer enn halvparten av dagene

Nesten hver dag

Vansker med å sovne, sove uten avbrudd eller sovet for mye

Ikke i det hele tatt

Noen dager

Mer enn halvparten av dagene

Nesten hver dag

Følt deg trett eller slapp

Ikke i det hele tatt

Noen dager

Mer enn halvparten av dagene

Nesten hver dag

Dårlig matlyst eller å spise for mye

- Ikke i det hele tatt
- Noen dager
- Mer enn halvparten av dagene
- Nesten hver dag

Vært misfornøyd med deg selv eller følt deg mislykket – eller følt at du har sviktet deg selv eller familien din

- Ikke i det hele tatt
- Noen dager
- Mer enn halvparten av dagene
- Nesten hver dag

Vansker med å konsentrere deg om ting, slik som å lese avisen eller se på TV

- Ikke i det hele tatt
- Noen dager
- Mer enn halvparten av dagene
- Nesten hver dag

Beveget deg eller snakket så langsomt at andre kan ha merket det. Eller motsatt - følt deg så urolig eller rastløs at du har vært mye mer i bevegelse enn vanlig

- Ikke i det hele tatt
- Noen dager
- Mer enn halvparten av dagene
- Nesten hver dag

Tanker om at det ville vært bedre om du var død eller om å skade deg selv

- Ikke i det hele tatt
- Noen dager
- Mer enn halvparten av dagene
- Nesten hver dag

Del 3 - Nedenfor finner du **seks spørsmål** knyttet til **søvn og tretthet**. Velg svaralternativet som beskriver deg best.

I løpet av den siste måneden, hvor mange dager per uke har du...

Brukt mer enn 30 min for å sovne inn etter at lysene ble slukket

- 0
- 1
- 2
- 3
- 4
- 5
- 6

7

Vært våken mer enn 30 min innimellom søvnen

0
1
2
3
4
5
6
7

Våknet mer enn 30 min tidligere enn du har ønsket uten å få sove igjen

0
1
2
3
4
5
6
7

Følt deg for lite uthvilt etter å ha sovet

0
1
2
3
4
5
6
7

Vært søvnig/trett at det har gått ut over skole/jobb eller privatlivet

0
1
2
3
4
5
6
7

Vært misfornøyd med søvnen din

0

1
2
3
4
5
6
7

Del 4 - Nedenfor finner du **to spørsmål** knyttet til **smerter**. Velg svaralternativet som beskriver deg best.

Hvor ofte har du opplevd følgende smerter i løpet av den siste måneden?

Hodepine

Ikke i det hele tatt
Noen dager
Mer enn halvparten av dagene
Nesten hver dag

Muskelsmerter (eks. rygg, skulder, nakke)

Ikke i det hele tatt
Noen dager
Mer enn halvparten av dagene
Nesten hver dag

4) Din bruk av reseptfrie midler

Siste del er knyttet til din bruk av **reseptfrie midler** i løpet av **den siste måneden** med fokus på følgende tre grupper: **beroligende midler, sovemidler og smertestillende midler.**

Du kan nå snu mobilskjermen tilbake til portrettmodus.

Har du brukt noen av følgende reseptfrie beroligende midler, sovemidler eller smertestillende midler?

Du kan velge flere alternativer.

Lunixen
Pascoflair
Sedix
Valerina Forte
Valerina Natt
Midler som inneholder melatonin
Aspirin
Fenazon-koffein
Paracet-midler
NSAIDs (Ibux, Naproxen, Diclofenac gel, Voltarol gel)

Nettskjema

Andre

Nei, jeg har ikke brukt reseptfrie beroligende midler, sovemidler og smertestillende midler.

Hvis andre, vennligst oppgi her.

Dette elementet vises kun dersom alternativet «Andre » er valgt i spørsmålet «Har du brukt noen av følgende reseptfrie beroligende midler, sovemidler eller smertestillende midler?»

Hvor har du kjøpt midlene?

Dette elementet vises kun dersom alternativet «Midler som inneholder melatonin eller Lunixen eller Valerina Forte eller Valerina Natt eller Sedix eller Pascoflair eller Paracet-midler eller NSAIDs (Ibux, Naproxen, Diclofenac gel, Voltarol gel) eller Aspirin eller Fenazon-koffein eller Andre » er valgt i spørsmålet «Har du brukt noen av følgende reseptfrie beroligende midler, sovemidler eller smertestillende midler?»

Du kan velge flere alternativer.

Apotek

Nettapotek

Helsekostbutikk (eks. Sunkost, Life)

Dagligvarebutikk

Nettbutikk

Andre

Hvis andre, vennligst oppgi her.

Dette elementet vises kun dersom alternativet «Andre » er valgt i spørsmålet «Hvor har du kjøpt midlene?»

Hva slags plager har du brukt midlene for?

Dette elementet vises kun dersom alternativet «Midler som inneholder melatonin eller Lunixen eller Valerina Forte eller Valerina Natt eller Sedix eller Pascoflair eller Paracet-midler eller NSAIDs (Ibux, Naproxen, Diclofenac gel, Voltarol gel) eller Aspirin eller Fenazon-koffein eller Andre » er valgt i spørsmålet «Har du brukt noen av følgende reseptfrie beroligende midler, sovemidler eller smertestillende midler?»

Du kan velge flere alternativer.

Hodepine

Muskelsmerter

Uro

Bekymring

Nervøsitet

Irritabilitet

Innsovningsvansker

Søvnforstyrrelser

Andre

Hvis andre, vennligst oppgi her.

Dette elementet vises kun dersom alternativet «Andre» er valgt i spørsmålet «Hva slags plager har du brukt midlene for?»

Hvor ofte har du brukt midlene?

Dette elementet vises kun dersom alternativet «Midler som inneholder melatonin eller Lunixen eller Valerina Forte eller Valerina Natt eller Sedix eller Pascoflair eller Paracet-midler eller NSAIDs (Ibux, Naproxen, Diclofenac gel, Voltarol gel) eller Aspirin eller Fenazon-koffein eller Andre » er valgt i spørsmålet «Har du brukt noen av følgende reseptfrie beroligende midler, sovemidler eller smertestillende midler?»

Av og til

Månedlig basis

Ukentlig basis

Daglig basis

Har du vært opptatt av riktig dosering av slike midler?

Dette elementet vises kun dersom alternativet «Midler som inneholder melatonin eller Lunixen eller Valerina Forte eller Valerina Natt eller Sedix

eller Pascoflair eller Paracet-midler eller NSAIDs (Ibux, Naproxen, Diclofenac gel, Voltarol gel) eller Aspirin eller Fenazon-koffein eller Andre » er valgt i spørsmålet «Har du brukt noen av følgende reseptfrie beroligende midler, sovemidler eller smertestillende midler?»

Dosering = hvor mye, hvor ofte og på hvilken måte middelen skal tas.

- Ja
- Nei

Hvilke informasjonskilder har du brukt for å vite doseringen til midlene?

Dette elementet vises kun dersom alternativet «Midler som inneholder melatonin eller Lunixen eller Valerina Forte eller Valerina Natt eller Sedix eller Pascoflair eller Paracet-midler eller NSAIDs (Ibux, Naproxen, Diclofenac gel, Voltarol gel) eller Aspirin eller Fenazon-koffein eller Andre » er valgt i spørsmålet «Har du brukt noen av følgende reseptfrie beroligende midler, sovemidler eller smertestillende midler?»

Du kan velge flere alternativer.

- Apotekansatt
- Pakning/pakningsvedlegg
- Min egen akademisk kunnskap
- Internett
- Familie/venner
- Andre

Hvis andre, vennligst oppgi her.

Dette elementet vises kun dersom alternativet «Andre» er valgt i spørsmålet «Hvilke informasjonskilder har du brukt for å vite doseringen til midlene?»

Hvordan ble du kjent med midlene?

Dette elementet vises kun dersom alternativet «Midler som inneholder melatonin eller Lunixen eller Valerina Forte eller Valerina Natt eller Sedix eller Pascoflair eller Paracet-midler eller NSAIDs (Ibux, Naproxen, Diclofenac gel, Voltarol gel) eller Aspirin eller Fenazon-koffein eller Andre » er valgt i spørsmålet «Har du brukt noen av følgende reseptfrie beroligende midler, sovemidler eller smertestillende midler?»

Du kan velge flere alternativer.

- Apotek
- Kostholdsbutikk
- Dagligvarebutikk
- Reklame
- Anbefalt av en bekjent
- Anbefalt av lege
- Andre

Hvis andre, vennligst oppgi her.

Dette elementet vises kun dersom alternativet «Andre » er valgt i spørsmålet «Hvordan ble du kjent med midlene?»

Hvorfor valgte du å bruke midlene?

Dette elementet vises kun dersom alternativet «Midler som inneholder melatonin eller Lunixen eller Valerina Forte eller Valerina Natt eller Sedix eller Pascoflair eller Paracet-midler eller NSAIDs (Ibux, Naproxen, Diclofenac gel, Voltarol gel) eller Aspirin eller Fenazon-koffein eller Andre » er valgt i spørsmålet «Har du brukt noen av følgende reseptfrie beroligende midler, sovemidler eller smertestillende midler?»

Du kan velge flere alternativer.

- Rask lindring
- Tidssparende fremfor å oppsøke legen
- Plager var ikke alvorlige nok for å oppsøke legen
- Høye kostnader ved å oppsøke legen
- Andre

Hvis andre, vennligst oppgi her.

Dette elementet vises kun dersom alternativet «Andre» er valgt i spørsmålet «Hvorfor valgte du å bruke midlene?»
